# A Rare Case of Paratesticular Leiomyosarcoma

**DOI:** 10.1155/2014/715395

**Published:** 2014-03-11

**Authors:** Shankar Haran, Vikram Balakrishan, Greg Neerhut

**Affiliations:** Urology Department, The Geelong Hospital, Ryrie Street, Geelong, VIC 3220, Australia

## Abstract

Paratesticular leiomyosarcomas are rare and can lead to significant morbidity and mortality, if inadequately diagnosed or treated. We present a case of paratesticular leiomyosarcoma in an 88-year-old man presenting with a left scrotal mass with ultrasound revealing that the mass is extratesticular. Left radical orchidectomy was performed and pathological examination of the resected specimen confirmed the diagnoses of high grade leiomyosarcoma with surgical margins clear of tumour. The patient was free of metastatic disease on further imaging and has been disease-free for 18 months. A review of the literature regarding paratesticular leiomyosarcoma presentation, diagnosis, and treatment is also discussed.

## 1. Introduction 

We report the first documented case of paratesticular leiomyosarcoma diagnosed and treated in Australia. A review of the literature regarding paratesticular leiomyosarcoma presentation, diagnosis, and treatment is also discussed.

## 2. Case

An 88-year-old male presented to his urologist with a tender swelling of his left testicle. There were no associated lower urinary tract or constitutional symptoms. His past history included prostate adenocarcinoma diagnosed 13 years prior to presentation, for which he had undergone transurethral resection of the prostate and regular monitoring. Physical examination revealed a 2 cm swelling arising from the upper pole of the left testicle. The mass was subcutaneous, firm, and tender. Scrotal ultrasonography identified a left sided hydrocele as well as an altered area of echogenicity at the upper pole of the left testis extending into the epididymis measuring approximately 15 mm in diameter. Prostate specific antigen was unchanged from previous measurements.

**Figure 1:**
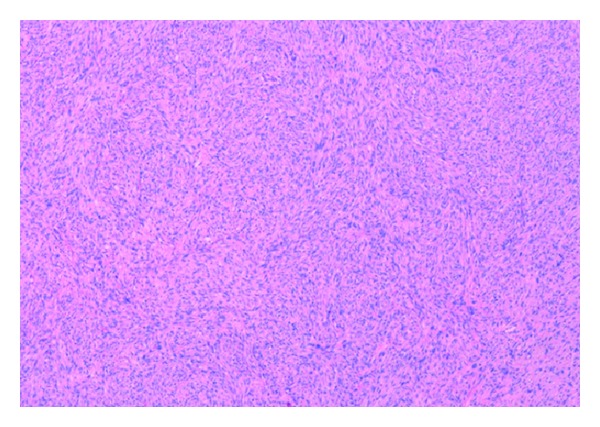
Interlacing fascicles of spindle cells.

**Figure 2:**
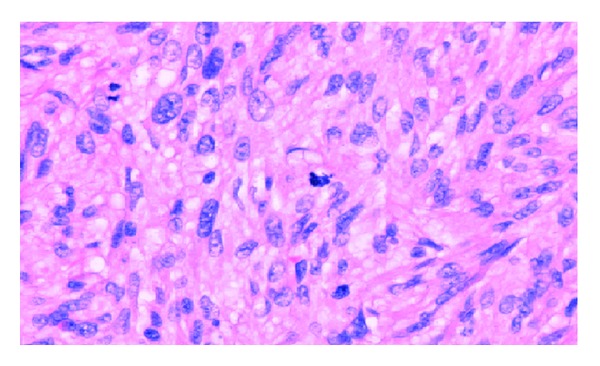
Spindle cells with typical cigar-shaped nuclei and abnormal mitotic figures.

**Figure 3:**
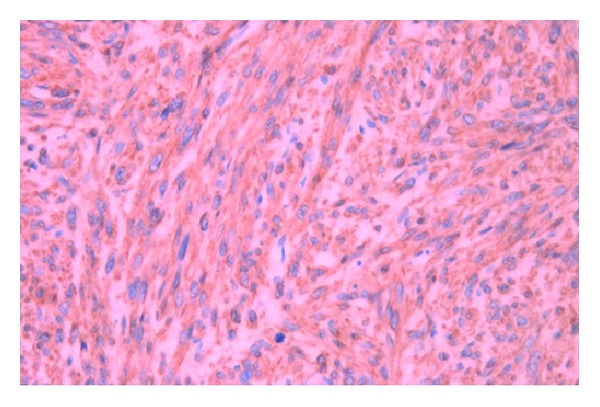
Tumour cells with diffuse positive staining for actin.

The patient underwent an elective left radical orchidectomy. The surgical specimen weighing 75 g consisted of testis, epididymis, and spermatic cord. It contained a 35 × 30 × 26 mm firm mass centred on paratesticular tissues adjacent to the upper pole of the left testis extending into the epididymis with a grey/white cut surface. Histologically, the tumour consisted of interweaving fascicles of cytologically malignant spindle shaped cells (Figure 1) with frequently bizarre nuclei. Abnormal mitotic figures were scattered throughout the lesion (Figure 2) and focal tumour necrosis was identified. The radial and spermatic cord resection margins were within normal tissue. Immunohistochemical stains were strongly positive for vimentin and smooth muscle actin (Figure 3). Histological features were consistent with a diagnosis of high grade leiomyosarcoma. Chest radiography and computed tomography of the abdomen and pelvis revealed no metastatic disease, and the patient has remained disease-free for 18 months after surgery.

## 3. Discussion

Soft tissue sarcomas of the genitourinary tract are rare. The American Cancer Society estimates that there were 11,280 new cases of soft tissue sarcoma diagnosed in the United States in 2012, accounting for less than 1% of all new cancer cases [1]. Approximately 2.1% of these cases will be localised to the genitourinary tract [2]. Paratesticular sarcomas are extremely rare with most masses of the scrotal sac localising to the testis and being neoplastic in nature [3]. The paratesticular region comprises the spermatic cord, testicular tunics, epididymis, and vestigial remnants such as the appendices epididymis and testis. Neoplasms arising from this region form a heterogeneous group with distinct behavioural patterns [4]. About 24% of spermatic cord tumours are leiomyosarcomas [5]. Around 110 cases of leiomyosarcoma of the spermatic cord have been reported in the literature [6] and only a handful of cases of the rarer epididymal leiomyosarcoma. The more common spermatic cord type arises from the smooth muscle cells of mesenchymal origin of the vas deferens, cremasteric muscle, and from arterial walls, while epididymal leiomyosarcomas arise from the smooth muscle surrounding the basement membrane of the epididymal tubule. Scrotal leiomyosarcoma is thought to arise from the dartos layer of the scrotum [7]. Like other sarcomas, leiomyosarcoma tends to infiltrate local tissues. Lymphatic spread may involve the external iliac, hypogastric, common iliac, and retroperitoneal lymph nodes while haematogenous metastases are primarily pulmonary [6, 8]. The vas deferens can act as a conduit allowing local spread to the scrotum, inguinal canal, or pelvis [6]. The behaviour of leiomyosarcoma is related to the site, size (particularly in areas where anatomical constraints limit adequacy of resection), histological grade, and presence of nodal or distant metastases [9, 10]. The American Joint Committee on Cancer (AJCC) classifies spermatic cord leiomyosarcoma as deep tissue. The presence of mitotic activity, percentage of necrosis, and severity of nuclear pleomorphism are all evaluated to grade the disease [11].

Peak incidence is in the sixth and seventh decade [7]. Typical clinical presentation is of a painless, firm, slow-growing, intrascrotal mass with palpation usually revealing the mass to be well defined, lobulated, mobile, and sometimes associated with a small hydrocele [8]. Work-up should include ultrasonography which is the primary imaging method for any cord or scrotal abnormality, with a sensitivity of 95–100% for differentiating intratesticular from extratesticular lesions [12]. A solid, heterogeneous mass is usually identified [13] with irregular, often increased vascularity on colour Doppler [14]; however, histological analysis of tissue is required for diagnosis. Typical histological findings include perpendicularly organised spindle cells with fascicular arrangement at low power and eosinophilic cytoplasm containing longitudinal fibrils and hyperchromatic blunt-ended nuclei at high power [15, 16]. The immunohistochemical profile of a leiomyosarcoma will reveal characteristics of smooth muscle differentiation including expression of smooth muscle actin and muscle specific actin and desmin. The expression of CD34 and cytokeratin has also been reported in some cases [17].

Due to the limited number of cases of this rare malignancy, an ideal treatment protocol has yet to be established with most documented treatments for paratesticular leiomyosarcoma grouped with those for other paratesticular sarcomas. The standard primary treatment is radical orchidectomy with high ligation of the spermatic cord and wide local resection of all nonvital structures. However, due to anatomical constraints, wide circumferential resection margins are rarely achieved and locoregional recurrence after definite surgery is common, occurring with a frequency of approximately 30–50% [10, 18–21]. Therefore, aggressive surgical strategies are advocated involving wide* en bloc *excisions of all potentially contaminated surrounding soft tissues aiming to obtain negative margin status as well as performing wide inguinal re-resection of soft tissue and scar excision in patients found to have inadequately resected disease [21]. If scrotal skin is involved, hemiscrotectomy is indicated [13]. Currently there is no clear indication for prophylactic lymphadenectomy for paratesticular leiomyosarcoma. Although previous reports of paratesticular sarcoma have identified regional nodal failure rates to be as high as 29% [22], there is no convincing evidence that leiomyosarcomas have such a predilection and the general consensus in the literature is that paratesticular leiomyosarcomas rarely involve locoregional lymph nodes rather spreading most frequently by direct extension. Furthermore, there are no available studies demonstrating that prophylactic lymph node dissection provides significant survival or recurrence benefit for patients with paratesticular leiomyosarcoma.

There is some evidence supporting the use of adjuvant radiotherapy for paratesticular sarcomas [20, 23, 24]. In a series of 21 cases, Catton and colleagues noted a 5-year disease-free survival of 58% with surgery alone and 100% with the addition of adjuvant radiotherapy (*P* < 0.01) [24]. A study from Massachusetts confirmed these results in a series of 18 patients with five of nine patients (56%) treated with surgery alone developing locoregional failure, whilst there were no cases of locoregional recurrence amongst the nine patients treated with both surgery and radiation [20]. It should be noted, however, that median follow-up for the irradiated group was shorter (63 versus 123 months) which may have led to an artificially increased rate of recurrence in the non-irradiated group. Despite these findings, there have been no studies on the use of radiotherapy in leiomyosarcoma specifically. In the case presented, given the age of the patient, clear surgical resection margins, and patient wishes, radiotherapy was not offered.

There is currently no clear role for adjuvant chemotherapy in the treatment of paratesticular leiomyosarcoma. A meta-analysis of 14 randomised trials of sarcomas at various sites showed that doxorubicin-based adjuvant chemotherapy led to an improvement in time to local and distant failure [25]. A trend toward overall improved survival was also noted, however, this was not statistically significant. Furthermore, Woll et al., in the largest Phase III randomized control trial to date, failed to show an improvement with chemotherapy for resected soft tissue sarcoma [26]. A single recent case study of a grade III paratesticular leiomyosarcoma showed an encouraging outcome for the role of systemic chemotherapy in addition to orchidectomy. Systemic chemotherapy consisted of nine cycles of ifosfamide and Adriamycin and neither local recurrence nor distant metastases occurred during the short follow-up period of 12 months after completion [27]. A lack of longer term follow-up and the relative paucity of such cases in the literature make interpretation of these results difficult.

## 4. Conclusion

Leiomyosarcoma should be considered as a differential diagnosis in any elderly male presenting with an intrascrotal mass. Although primary management has previously been based on radical orchidectomy with high ligation of the spermatic cord, locoregional recurrence rates are as high as 50%. Adjuvant radiation therapy can lead to improved locoregional control and has a role in patients with nonmetastatic paratesticular leiomyosarcoma. The limited number of cases of this rare disease as well as the inconsistent management strategies utilised requires that further research be performed to formulate an ideal treatment protocol.
